# ‘It just has to click’: Internists’ views of: what constitutes productive interactions with chronically ill patients

**DOI:** 10.1186/s12913-016-1430-6

**Published:** 2016-05-27

**Authors:** N. M. H. Kromme, C. T. B. Ahaus, R. O. B. Gans, H. B. M. van de Wiel

**Affiliations:** Division of Chronic and Vascular Disease, University of Groningen, University Medical Centre, Hanzeplein 1, 9751 RB Groningen, The Netherlands; Faculty of Economics and Business, Centre of Expertise Healthwise, University of Groningen, University Medical Centre Groningen, Nettelbosje 2, 9747 AE Groningen, The Netherlands; Department of Internal Medicine, University of Groningen, University Medical Centre Groningen, 9751 RB Groningen, The Netherlands; Wenckebach Institute, University of Groningen, University Medical Centre Groningen, Hanzeplein 1, 9751 RB Groningen, The Netherlands

**Keywords:** Productive patient–physician interaction, Chronic Care Model, Goal orientations, Subspecialty, Medical context, Rapport

## Abstract

**Background:**

According to the Chronic Care Model, productive interactions are crucial to patient outcomes. Despite productive interactions being at the heart of the Model, however, it is unclear what constitutes such an interaction. The aim of this study was to gain a better understanding of physician views of productive interactions with the chronically ill.

**Method:**

We conducted a qualitative study and interviewed 20 internists working in an academic hospital. The data were analyzed using a constructivist approach of grounded theory. To categorize the data, a coding process within which a code list was developed and tested with two other coders was conducted.

**Results:**

The participants engaged in goal-directed reasoning when reflecting on productive interactions. This resulted in the identification of four goal orientations: (a) health outcome; (b) satisfaction; (c) medical process; and (d) collaboration. Collaboration appeared to be conditional for reaching medical process goals and ultimately health outcome and satisfaction goals. Achieving rapport with the patient (‘clicking,’ in the term of the participants) was found to be a key condition that catalyzed collaboration goals. Clicking appeared to be seen as a somewhat unpredictable phenomenon that might or might not emerge, which one had to accept and work with. Goal orientations were found to be related to the specific medical context (i.e., a participant’s subspecialty and the nature of a patient’s complaint).

**Conclusions:**

The participants viewed a productive interaction as essentially goal-directed, catalyzed by the two parties clicking, and dependent on the nature of a patient’s complaint. Using the findings, we developed a conceptual process model with the four goal orientations as wheels and with clicking in the center as a flywheel. Because clicking was viewed as important, but somewhat unpredictable, teaching physicians how to click, while taking account of the medical context, may warrant greater attention.

**Electronic supplementary material:**

The online version of this article (doi:10.1186/s12913-016-1430-6) contains supplementary material, which is available to authorized users.

## Background

To help meet the needs of the chronically ill, the McColl Institute for Healthcare Innovation developed the Chronic Care Model (CCM). The aim was to improve the quality of care and outcomes of the chronically ill through transforming the healthcare system [1, 2]. In the Model, community resources and healthcare organizations support four main elements (self-management support, delivery system design, decision support, and clinical information systems). These elements contribute to ‘productive interactions’ between an informed, activated (engaged) patient and a prepared, proactive physician and/or practice team [1].

Despite the centrality of productive interactions to patient outcome [3], this concept, at the heart of the CCM, has surprisingly not been adequately clarified [4]. Research on CCM has focused mainly on the effectiveness of the aforementioned four elements in achieving the desired outcomes [5]. This is all the more remarkable because patients regularly perceive that their needs, expectations, and preferences regarding information exchange, shared decision making, interpersonal interactions and self-care support are not met in their interactions with physicians [6–10]. From their side, physicians experience difficulties, uncertainties, fatalism, and/or a high workload in interacting with chronically ill patients [11–15].

Our primary aim was to clarify the concept of productive interaction. While there is a stream of scientific articles on ‘patient-centered’ interaction, we failed to find any theoretical or empirical studies investigating what constitutes a ‘productive’ interaction in a medical encounter. In attempting to fill this gap, we were particularly interested in the views of physicians. Their role as a communicator is to facilitate the ‘dynamic exchanges’ that occur within the medical encounter [16]. However, interventions aimed at improving the communication skills of physicians seem to deliver small and often disputed effects, especially as regards medical specialists [17]. In this paper, we explore the views of internists on their ‘productive interactions’ with patients with chronic conditions, based on the following questions:

- What do internists view as a productive interaction with patients with chronic conditions?
- Can a conceptual model of a productive interaction be derived from their views?

## Methods

### Design and participants

A qualitative study was designed and conducted to explore internists’ views of productive interactions. We focused on internists because the Internal Medicine specialty treats many various chronic diseases. We carried out an in-depth investigation into how internists describe their ideas, images, expectations, and perceptions of a productive interaction, and searched for detectable patterns. The breadth and depth of their professional training enables them to deal with the complex problems of chronically ill patients, both as generalists and as specialists [18]. This choice further reduced the need to involve other medical specialties in this study.

We focused on internists working in the Department of Internal Medicine at a university medical center. To create some variation within the sample, we selected equal numbers of generalists and subspecialists, and subsequently included a broad range of participants in terms of their gender, age, and experience. Such variation enables generation of themes, relationships, and hypotheses from collected data, and the capacity to compare subgroups within the overall sample [19, 20]. The selected generalists from the General Internal Medicine and the Elderly Medicine/Geriatrics departments could be characterized by their focus on diagnostics and acute care. The selected Endocrinology and Nephrology subspecialists also have a diagnostic role, but are more focused on chronic care. Consequently, the relationships with patients tend to differ in the two groups, with shorter-term associations for generalists and interactions spread over a longer period for subspecialists. We expected the internists to have somewhat similar views on productive interactions because people generally share views when they share a professional background; however, we also anticipated that views would differ as a result of differing experience with the patient groups in the respective subspecialties [21].

Between October 2011 and April 2012, we interviewed 20 internists who met our selection criteria (see Table 1). Participants included internists who had responded directly to our request for participants, plus others we individually approached to satisfy our sampling requirements. We stopped seeking out and interviewing new participants when new issues were no longer arising in the interviews, based on the argument that variation and saturation are the most relevant criteria for validating a sample [22].

**Table 1:** Characteristics of the participants

Characteristics	Number included (% of total staff)
Sub-discipline:	
Generalists:	10 (26 %)
Elderly medicine/Geriatrics	4 (11 %)
General internal medicine	6 (16 %)
Subspecialists:	10 (26 %)
Endocrinology	5 (13 %)
Nephrology	5 (13 %)
Gender:	
Men	12 (32 %)
Women	8 (21 %)
Age (and gender division):	
34–41 years (5 women, 4 men)	9 (24 %)
45–61 years (3 women, 8 men)	11 (29 %)

### Data gathering and analysis

The semi-structured interviews started with our briefly explaining the CCM and posing some general questions about the interviewee’s background and interest in medicine. The interviewer (the first author) then guided the interviews using a short topic list.

Topics covered were the definition, course and outcome expectations of a productive interaction; influential factors; means of engaging with patients; and the competencies needed to interact productively (see Additional file 1). The topics were introduced in a flexible way, and the interviews took the form of natural conversations. The interviewer regularly paraphrased the interviewee’s responses to check that she had understood them [23]. If new and relevant issues arose, these were then included in subsequent interviews. The interviews lasted from 44 to 108 min, were audio recorded and transcribed with the verbal informed consent of the interviewees. Transcripts of the interviews were imported into Atlas.ti software program (version 5.2.18, Atlas.ti Scientific Software Development GmbH, Berlin) to assist in project management and data analysis. Interview transcripts were fully coded, resulting in a total of 867 coded or labeled fragments (36 to 60 fragments per interview).

We analyzed the data using a constructivist approach to grounded theory. This approach involves a step-by-step coding process in which one makes hermeneutic sense of the data [20, 24] and compares each interview with all the others in order to identify patterns or themes. The first stage of the analysis involved the lead author in an open, initial coding procedure [20] that resulted in an initial list of codes corresponding closely to the text fragments extracted from the first six interviews. After discussing this list with a second and third coder, the first six interviews were recoded and new interviews coded using the adapted list. As this process progressed, the code list was further refined, followed by further testing and discussion to establish the most relevant codes for labeling an interaction as ‘productive.’ This process identified a ‘productive interaction’ category that comprised 15 different codes and 275 fragments that could be attached to these codes. Subsequently, we made so-called ‘thick descriptions’ (i.e., descriptions of the depth, breadth, context, and nuances) of those text fragments that had been given codes related to productive interactions [24]. In the final analytical stage, we further reduced the data through a process of displaying them in matrices. Here, we identified the goal orientations within which the participants’ descriptions could be placed [25]. After this, we derived an initial conceptual model through an iterative process of searching in the related literature and in the data [20].

Internal validation was established by systematically verifying the findings in the data and by extensively discussing the findings with an oversight research group representing different perspectives and disciplines (i.e., anthropology, healthcare and quality management, internal medicine and health psychology).

### Ethical review

The Committee for Medical Ethics of the University Medical Centre Groningen (METcUMCG) granted an exemption from the requirement of ethical approval of this study according to the Dutch ‘Medical Research Involving Human Subjects Act (WMO).’ Our research adhered to the standards as described within the Qualitative Research Review Guidelines (RATS) (http://www.equator-network.org/reporting-guidelines/qualitative-research-review-guidelines-rats/).

## Results

### Goal orientations of a productive interaction

We found that the reasoning of the participants about productive interactions was goal-directed. We could subdivide their intentions into four goal orientations: (a) health outcome, (b) satisfaction, (c) medical process, and (d) collaboration. It also appeared that some goals were more frequently mentioned or emphasized than others, depending on the subspecialty of the participant and on the medical context. The subspecialists (endocrinology and nephrology) tended to make reference to patients with medically clear (i.e., specific) complaints related to a diagnosed chronic disease (e.g., diabetes or chronic kidney disease). The generalists (elderly medicine/geriatrics and general internal medicine), in contrast, more often referred to patients with medically vague complaints, and in particular to patients with unexplained but chronic complaints (see Table 2).

**Table 2:**
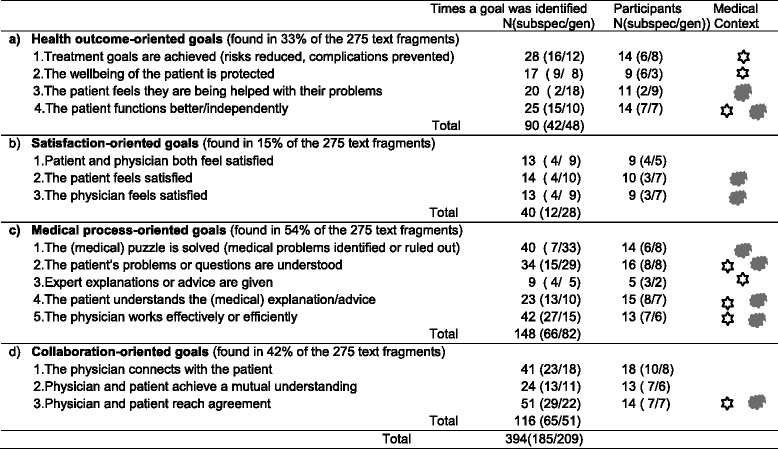
Frequency of goals, types of participants, and relationship to the medical context

Goal orientations of the participants based on 275 selected text fragments

The first column of values shows how often we identified a goal in the selected text fragments of either the subspecialists (subspec) or the generalists (gen). The second column shows the number of subspecialists and generalists contributing these text fragments. The third column indicates whether the medical context the participants referred to was - medically clear (specific) complaints and/or a diagnosed condition (marked with a ) - vague, medically unexplained (nonspecific) complaints (marked with a )

#### Health outcome-oriented goals (a)

Achieving health outcome goals was seen as one result of a productive interaction. The participants related two such goals primarily to patients with a diagnosed condition (e.g., diabetes or hypertension) and/or specific complaints. First, treatment goals are achieved (risks reduced, complications prevented) generally reflected achievement of a desirable clinical outcome, as conveyed in this comment:

‘A productive interaction is, in my terms, very much targeted at the final product, in particular good blood pressure, good average sugar regulation, and good cholesterol levels, and … this is how you can measure whether something is productive or not.’ (Subspecialist)

Second, participants referred to the goal of ‘the wellbeing of the patient is protected,’ meaning that they balanced achieving medical goals against the overall benefits for the chronically ill elderly patients.

The third goal, ‘the patient feels they are being helped with their problems,’ was, however, mostly associated with nonspecific complaints. In these cases, it was mainly the generalists who seemed to reflect skepticism about being able to genuinely support such patients. Finally within this category, the goal that ‘the patient functions better/independently’ had different meanings depending on the nature of a patient’s condition. In the case of a diagnosed chronic condition, the participants associated the ‘patient functions better/independently’ goal with the patient’s ability to self-care/self-manage, because of the expected benefits this had for the patient’s life expectancy. However, in the case of vague medically unexplainable (nonspecific) complaints, ‘the patient functions better/independently’ was primarily associated with increasing the patient’s insight into their complaints. It was also seen as meaning that patients could ‘get on with their lives,’ turning their attention away from medical explanations for their ailments.

#### Satisfaction-oriented goals (b)

Both parties’ achieving a general feeling of satisfaction was viewed as an important result of a productive interaction. This is reflected in the goal that ‘the patient and the physician both feel satisfied.’ It included being satisfied with both ‘the whole process of adapting medical treatment to the patient’ and ‘interacting with the patient.’ Some warned that successfully fulfilling the medical task does not guarantee a satisfied patient. Others, such as this interviewee, pointed to the essential role of collaboration in achieving satisfaction:

‘It is the collaboration between patient and doctor that makes both leave the room feeling satisfied. That is a real productive interaction. The basic condition for that to occur is that you try to help someone as much as it is medically possible and that the patient feels helped—that the patient has the sense that they have been heard, and that you have the feeling that there has been an authentic contact and not merely an instrumental contact.’ (Generalist)

The generalists emphasized ‘the patient is satisfied’ and ‘the physician is satisfied’ goals more often than did the subspecialists. They mainly related these goals to patients with medically unexplained complaints. Solving these patients’ problems was generally viewed as difficult and associated with the physician’s insecurities when a medical cause was not found. The ‘physician feels satisfied’ goal appeared to mean different things depending on the nature of a patient’s condition. Where a medical cause was not found, the participants expressed a sense of feeling rewarded for their efforts when a patient seemed better able to cope with the complaints, especially because several participants mentioned they felt tired and sometimes exhausted by supporting such patients. When there was a diagnosed condition, being ‘satisfied as a doctor’ seemed more often associated with being proud of a patient’s improved functioning and/or positive health outcomes.

#### Medical process-oriented goals (c)

Participants often expressed medical process-oriented goals, and pointed to what they found productive based on experiences in their daily practices. This resulted in a mixed image of medical process goals, related to a participant’s specialty and the condition of their patients.

Although the ‘solving the (medical) puzzle’ goal refers to the diagnostic task in general, this goal was most often expressed by generalists as ‘ruling out a medical cause’ in the context of vague (nonspecific) complaints. Second, the goal that ‘the patient’s problems or questions are understood’ was generally viewed as important. This goal was more often expressed by the generalists than the subspecialists, particularly when discussing the extent to which they should delve deeper into the patient’s background and problems in the case of problems such as chronic fatigue. The third goal, ‘expert explanations or advice are given,’ was primarily (but not often) seen as relevant in the context of patients with a diagnosed condition. Participants often related the fourth goal, ‘the patient understands the (medical) explanations/advice,’ to a patient's’ adherence to the proposed treatment where there was a diagnosed condition:

‘A productive interaction is one where they understand what I am going to do, and when I have made a diagnosis (or not), what that means—and when I prescribe pills, that they have to take them.’ (Generalist)

For patients with medically unexplained complaints, this goal meant the patient’s understanding that further searching for medical explanations for their ailments would not be helpful.

‘The conversation is successful when, after three-quarters of an hour, I see that the penny has really dropped, when the person says that they have gained something from it, and that they really see that they do not have to visit 50 other doctors to get the answer.’ (Generalist)

The fifth goal within this orientation, that ‘the physician works effectively or efficiently,’ was often expressed in the context of the time available, or as a balance of achieving consultation objectives and finishing within the allotted time. Several subspecialists emphasized that they lacked the time needed to ‘work effectively,’ i.e., to talk to a patient about their condition, as well as to discuss laboratory results and treatment options. They viewed this as important in reducing risks irrespective of the time and costs involved. When considering patients with nonspecific complaints, several generalists stressed the need to ‘work efficiently’ while also frequently mentioning that they often invested time in exploring and discussing problems.

#### Collaboration-oriented goals (d)

Achieving collaboration-oriented goals was often mentioned as an essential part of a productive interaction. The first related goal, that ‘the physician connects with the patient,’ was associated with ‘getting in touch’ and ‘building the important bond of trust,’ with the latter viewed as especially important to establish in the first contact. Several participants also stressed the importance of ‘investing in a personal safe atmosphere so that people do not think they are just a number.’ This could involve social talk, remembering the patient’s personal situation, and actually calling patients to give them laboratory results. The second goal, that ‘physician and patient achieve a mutual understanding,’ was viewed as essential for *‘*understanding the patient’s actual request for help.’ It was further defined as reaching a ‘real understanding,’ a process involving mutual openness and respect, as well as listening attentively and speaking comprehensibly:

‘A productive interaction is one where you create mutual openness, where you can understand each other in that way; not just hear, but really understand.’ (Subspecialist)

Several participants suggested that when a physician truly explores a patient’s problems, such that ‘the patient feels heard and understood,’ the patient will disclose more about their problems. Consequently, the physician will be better able to understand the patient’s concerns, more likely make the correct diagnosis, and be better able to give advice that the patient will follow. The third goal in this category, that ‘the physician and the patient reach agreement’ or ‘consensus,’ was expressed as ‘having the same goals’ or ‘being on the same wavelength’ and often associated with a focus on solving problems medically. However, the more it concerned patients in a somewhat stable chronically ill stage, the more reaching agreement was defined as the process of negotiating treatment options.

‘With the chronically ill, it is often about negotiating, in the sense that the outcome is satisfactory to both sides, and that you do not impose your will because that is not productive, because someone can say yes but can act no.’ (Subspecialist)

When it came to patients with medically unexplainable complaints, the participants more often talked about discussing procedures and/or about the need to change a patient’s perspective. Sample quotes of all goals can be found in Additonal file 2.

### A main condition: clicking with the patient

During the interviews, we observed that the term ‘clicking with the patient’ was often used by participants from all the subspecialties. As such, we identified it as a central condition for a productive interaction.

‘Sometimes you do sense a click, and sometimes you feel less of a click. It is a feeling you have as soon as they enter the room, how they look at you, you just see whether they trust you or not … I think the success factor is mainly determined by whether we click, and whether I strike the right chord, and sometimes that happens by chance.’ (Generalist)

The participants associated ‘clicking’ not only with connecting from the start, but also with exchanging information, registering nonverbal signs (such as anxiety or distress), and with reaching agreement or advancing the conversation in general.

‘A productive interaction to me is one where there is a click, that you are talking about the same things, and the conversation advances, that you both leave with good feelings.’ (Subspecialist)

Although some claimed that they invested in the relationship and/or in creating a safe atmosphere in order to develop a bond of trust, many participants described ‘clicking’ as a spontaneous phenomenon that simply happened. They saw clicking as automatically evoking positive feelings of empathy and trust, which made it easier to put themselves in the patient’s shoes. Although precisely defining ‘clicking’ appeared difficult for the participants, some tried to explain it as a sudden feeling based, for example, on a story or on transference. Further, participants associated ‘clicking’ with patients who were active, empowered, and communicative. Conversely, ‘not clicking’ was linked to the opposite traits and expressed as a patient’s behavior that ‘causes friction, after which nothing runs smoothly.’ The participants mentioned several patient behaviors that could cause friction in the form of irritation, annoyance, or even stress: from patients who complain about endless lists of ailments to patients who behave in demanding or manipulative ways or who are unreasonably angry. They also commented that it was difficult to click with patients who play ignorant, are taciturn, have psychiatric co-morbidity, or look uncared for and/or smell strongly. Some participants tried to put such negative feelings into perspective by saying these situations do not occur that often. One expressed the danger of stereotyping:

‘Of course you are also human; you cannot prevent some people getting more on your nerves than others. With some people, you know in advance that they will tell you a very long-winded story and that the answer to every question will be that they suffer from it. Usually, your feeling is correct but you need … to force yourself to be open-minded because that is part of your profession.’ (Generalist)

Nevertheless, most participants seemed to accept it as inevitable that they would get along better with some patients than with others. They expressed that they acted in a less open-minded and caring way, and were more businesslike, toward those with whom they failed to click:

‘With one person it clicks better than with another, and of course that is natural. People I do not click with tend to disappear from my outpatients list after a certain time … When it goes well, you are less businesslike.’ (Subspecialist)

### An initial conceptual model of a productive interaction

**Fig. 1:**
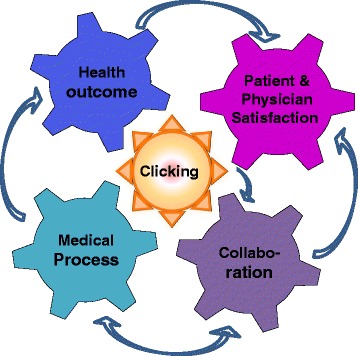
Participants’ model (view) of a productive interaction

Through this process, we uncovered four types of goal orientations that were related to the medical context and a central condition that constituted main elements of a productive interaction. Essentially, the participants understood ‘clicking’ as something that makes the whole process run smoothly. On this basis, we visualized the participants’ image of a productive interaction as a cyclic process with the goal orientations as gears and ‘clicking’ as the flywheel in the center (see Fig. 1).

The following is a more detailed conceptualization of how participants viewed the workings of a productive interaction. First, clicking with a patient leads to a mutual feeling of connectedness and trust, which enhances mutual understanding and/or the feeling of being on the same wavelength. Mutual understanding makes it more likely that the physician will explore a patient’s problems in a way that ‘the patient feels heard and understood’ and that the physician understands the patient’s problems and makes the right diagnosis. Further, the physicians expected both parties to have, or to come to, common goals and reach an understanding and agreement to accept the advice that was offered. This would increase the likelihood that both the patient and the physician would be satisfied with the collaboration, the medical process, and the advice offered; and that the patient would then act upon this advice. Finally, trust seemed to be viewed as a mediator between ‘clicking’ and the collaboration goals, whereas a lack of time and a patient’s limited ability to understand the explanations offered were viewed as inhibiting factors in achieving the goals of the medical process.

## Discussion

### Findings

In this study, we explored the views of 20 internists as to what constitutes a ‘productive interaction’ (the core concept of the CCM) and integrated their descriptions into a conceptual model of a productive interaction. Although we had anticipated that participants with a general focus would have different views from the specialists, the findings indicated that the views of both groups were similar. Physicians in both groups tended to define a productive interaction in terms of the same four goal orientations, and ‘clicking with the patient’ was generally viewed as a main condition. The participants mostly differed in how they talked about reaching goals for patients with medically clear versus medically unexplainable complaints. Before turning to a detailed discussion of our results, we introduce cultural model theory as a lens through which we will discuss their views. Cultural models or schemas or understandings refer to relatively stable cognitive structures ([21], p. 54). Within a social group, people will have collective understandings based on shared experiences and a shared identity. By applying this perspective we were able to recognize *collective understandings* that refer to the identity and meaning system of the participants as part of their medical profession [21].

First, we identified the shared principle of intentionality regarding achieving outcomes and satisfaction, as well as implementing the medical process and successfully collaborating with the patient. These intentions pertain to medical conduct and identity in general, as well as to *a collective, functionalist understanding* of the medical task based on professional standards [26, 27]. In order to cure and heal, physicians need to solve medical problems effectively, have answers to a patient’s complaints, and support the patient in coping with illnesses and relieving suffering. As such, they need to follow procedures for resolving problems efficiently, give clear treatment advice, interact humanely, and satisfy the patient [28]. In addition, external influences on the participants’ intentions were also recognizable. For example, in their goal orientations, the participants reflected their natural biomedical discourse as well as the current discourse on patient engagement, patients caring for themselves, and quality of life.

Second, we discerned, as a *collective understanding*, that the participants shared how they related a goal to the medical context. They generally divided the context into medically clear and explainable (specific) complaints, and medically vague and often-unexplainable (nonspecific) complaints. Patients with specific complaints were often considered seriously ill and needing the help of the physician. Solving the problems of these patients seemed a natural task, although not always easy, due to issues such as lack of time or the patient’s inability to understand the advice. Solving the problems of patients with nonspecific complaints often appeared to be an unrealistic and exhausting ambition. This division of patients into these two groups has been recognized elsewhere [29–31], with the latter chronically ill often labeled as patients with Medically Unexplained (Physical) Symptoms (MUS) [32, 33].

The third, and the central, *collective understanding* we found among the participants was that ‘clicking’ with the patient influences all the goals, as well as the initial intention to collaborate. By ‘clicking,’ the participants seemed to refer to something on the intuitive level, to transference and/or to a deeper existential dimension that establishes trust and makes it easier to empathize. In addition, several participants stressed the importance of a genuine contact as a fundamental basis for collaboration with a patient. It has been argued that people use particular statements, such as ‘we really clicked’ and ‘we had chemistry,’ to describe interpersonal interactions that go exceedingly well and to indicate good rapport [34, 35]. Rapport reflects the quality of an interaction between people, and is evaluated in terms of emotional positivity and as a perceived unity in the interaction [36], or as a whole that is more than the sum of the parts. As such, rapport is a nonverbal phenomenon, as well as a social construct that comprises the following components: *positive affect*, *mutual focus of attention*, and *interpersonal coordination.* Positive affect concerns the feeling of liking each other, which is associated with trust, mutual understanding, caring, and giving support. The other two components pertain to harmony, agreement, and accord, or to synergy in a genuine interaction [36, 37]. Finally, rapport is seen as an essential part of the therapeutic relationship and bond, and one that has a positive influence on the clinical outcome [38], although situations can differ [35] and changes are possible during the relationship [39]. Similarly, rapport is probably an important aspect of the ‘the whole and shared mind’ concept that Epstein recently introduced to enhance clinical decision-making [40]. Rapport, however, does not only exist in therapeutic relationships but is also part of non-therapeutic working alliances, friendships, and romantic relationships [34]. We also found this: participants used the term ‘clicking’ not merely in the medical context but also to give expression to a broader *collective understanding* of authentic relationships between people.

Our final remarks concern our model of a productive interaction. The four main elements of the CCM, and self-management support programs in particular, are broadly implemented in primary care in Western countries [41]. In the CCM, self-management support is seen as a condition needed to involve and activate patients in productive interactions, with collaborative management of the illness, and ultimately better health outcomes, as results [2, 42]. Wagner and others have argued that involving patients in their own care shows more clearly positive results on health outcome, as does improving attitudes and/or communication skills of physicians, which is often a focus of patient-centered approaches [42–45].

In our model, conversely, the participants viewed collaboration as conditional for reaching medical process goals, and ultimately, health outcome and satisfaction; and 'çlicking with the patient' was viewed as a spontaneously emerging phenomenon that catalyzes collaboration. Their view of collaboration also echoes the therapeutic relationship as earlier described in the work of Roger and Balint, and is consistent with the more recently developed relationship-centered models [46, 47]. In this view doctors do not stay neutral but create a bond needed for empathetic understanding of a patient’s problems as a person [48].

A central issue in our model is that the participants explained clicking as on the level of affect and as a fact of life, something that either happens or does not, and that one has to accept and work with in this reality. This view contradicts current models on patient-physician interaction, which imply that establishing rapport can be learned [16, 27, 49]. Moreover, the experienced difficulty of building a relationship with patients with medically unexplained symptoms seemed to justify lower levels of support and follow-up in such cases. However, the literature [50, 51], including guidelines [52], indicates that these patients would also benefit from a therapeutic relationship with physicians because, like persons with more easily treated conditions, they too have diverse and complex problems that impact quality of life [50–52].

Our findings contribute to a better understanding of productive interactions from the viewpoint of physicians. Creating awareness among physicians that rapport can be learned [53] and that the medical context; i.e., the nature of the complaint influences the process of building rapport [54, 55] may deserve greater attention within intervention and implementation programs of the CCM. Educators could use these findings in continuous medical education in primary care as well as in medical specialty care.

### Strengths and limitations

A strength of our research approach and conducting in-depth interviews in a natural way is that it enables one to derive shared intentions and collective understandings, and to construct a model [23, 24]. These understandings are likely to reflect broader groups of internists, and perhaps also of primary care physicians, because they basically share the same profession and largely the same patient groups [23]. The findings, however, should be validated for different contexts.

A basic issue is that what the participants understand as a professional productive interaction is not necessarily the same as what they experience or achieve in practice. Further, it is likely that gender, age, and experience, as well as individual beliefs or motives, will affect strategies and behaviors. Although we have not rigorously analyzed such differences, we did gain the impression that gender, and perhaps other aspects, do have an influence on participants’ interaction strategies or styles.

We should also caution that the ‘goal counts’ shown in Table 2 are not intended to imply the significance of the goals per se, but are offered as a closer look at the content of the text fragments. Nonetheless, we do believe that the relative frequencies with which the various goals were mentioned give some indication of their relative importance. Hence, the relative frequencies may function as a guide for further investigation.

To avoid recognition of colleagues in the interview data no one from the internal medicine department was involved in data analysis. However, inside knowledge of the internal medicine practice appeared to be essential for the interpretation and during the discussion of the findings. A risk is that an interviewer may not have been able to create a suitable interview climate or to establish sufficient rapport to get full and honest answers from the interviewees. However, several participants in our study commented positively on the interview process, so we do not see this as a major concern. Some participants mentioned that answering questions on productive interactions was a useful opportunity to talk about and reflect on their interactions with patients. Nevertheless, it is always possible that the interview setting influenced some answers, and that some participants may have found it difficult to provide full answers to unexpected questions. However, our approach is the only way to elicit the views and understandings of participants beyond a framework of predefined questions [23], and we believe that if conversations are conducted in a respectful, open, and natural way, with enough space to elaborate on thoughts that arise, this goal can be achieved.

## Conclusions

The participants viewed a productive interaction as one that was goal-directed, dependent on the nature of the patient’s complaint, and catalyzed by clicking with the patient. Although we had not expected it, we found that clicking, i.e., establishing rapport, played a central role in a productive interaction. Further, we saw that while clicking was viewed as important, it was also seen as somewhat unpredictable, something that may or may not happen. That is, the level of rapport with an individual patient seemed to be a fact of life, something one simply had to accept and work with. However, other academics have argued that establishing rapport is a teachable skill. Given that clicking seems beneficial for both the wellbeing of patients and their physicians, creating awareness among physicians that conditions conducive to clicking can be learned may warrant a place in the curriculum, as well as in programs implementing the CCM.

## Availability of data and materials

Confidentiality agreements prevent us from sharing the raw interview data.

## Additional files

Additional file 1: Interview topic list. (DOC 35 kb) Additional file 2: Sample quotes for all goals. (DOCX 24 kb)

## References

[1] Wagner EH (1998). Chronic disease management: what will it take to improve care for chronic illness?. Eff Clin Pract.

[2] Wagner EH, Austin BT, Davis C, Hindmarsh M, Schaefer J, Bonomi A (2001). Improving chronic illness care: translating evidence into action. Health Aff (Millwood).

[3] Cramm JM, Nieboer AP. A longitudinal study to identify the influence of quality of chronic care delivery on productive interactions between patients and (teams of) healthcare professionals within disease management programmes. BMJ Open. 2014;4(9): e005914-2014-005914. 10.1136/bmjopen-2014-005914 PMC4170203 25239294

[4] Oprea L, Braunack-Mayer A, Rogers WA, Stocks N (2010). An ethical justification for the Chronic Care Model (CCM). Health Expect.

[5] Davy C, Bleasel J, Liu H, Tchan M, Ponniah S, Brown A. Effectiveness of chronic care models: opportunities for improving healthcare practice and health outcomes: a systematic review. BMC Health Serv Res. 2015;15:194-015-0854-8. 10.1186/s12913-015-0854-8 PMC4448852 25958128

[6] Kiesler DJ, Auerbach SM (2006). Optimal matches of patient preferences for information, decision-making and interpersonal behavior: Evidence, models and interventions. Patient Educ Couns.

[7] Schattner A, Bronstein A, Jellin N (2006). Information and shared decision-making are top patients’ priorities. BMC Health Serv Res..

[8] Bastiaens H, Van Royen P, Pavlic DR, Raposo V, Baker R (2007). Older people’s preferences for involvement in their own care: A qualitative study in primary health care in 11 European countries. Patient Educ Couns.

[9] Mirzaei M, Aspin C, Essue B, Jeon YH, Dugdale P, Usherwood T, Leeder S. A patient-centred approach to health service delivery: improving health outcomes for people with chronic illness. BMC Health Serv Res. 2013;13:251-6963-13-251. 10.1186/1472-6963-13-251 PMC3706210 23819721

[10] Bensing J, Rimondini M, Visser A (2013). What patients want. Patient Educ Couns.

[11] Smith S (1995). Dealing with the difficult patient. Postgrad Med J.

[12] Becker G, Kaufman SR (1995). Managing an uncertain illness trajectory in old age: patients’ and physicians’ views of stroke. Med Anthropol Q.

[13] Loewe R, Schwartzman J, Freeman J, Quinn L, Zuckerman S (1998). Doctor talk and diabetes: Towards an analysis of the clinical construction of chronic illness. Soc Sci Med.

[14] Asbring P, Narvanen AL (2003). Ideal versus reality: physicians perspectives on patients with chronic fatigue syndrome (CFS) and fibromyalgia. Soc Sci Med.

[15] Hinchey SA, Jackson JL (2011). A cohort study assessing difficult patient encounters in a walk-in primary care clinic, predictors and outcomes. J Gen Intern Med.

[16] Frank J (2005). The CanMEDS 2005 physician competence framework.

[17] Wouda JC, van de Wiel HB (2013). Education in patient-physician communication: how to improve effectiveness?. Patient Educ Couns.

[18] Molleman E, Broekhuis M, Stoffels R, Jaspers F (2008). How health care complexity leads to cooperation and affects the autonomy of health care professionals. Health Care Anal.

[19] Glaser BG, Strauss AL (1966). Purpose and credibility of qualitative research. Nurs Res.

[20] Constructing CK, Theory G (2006). A Practical Guide Through Qualitative Analysis.

[21] Strauss C, Quinn N (1997). A Cognitive Theory of Cultural Meaning.

[22] Francis JJ, Johnston M, Robertson C, Glidewell L, Entwistle V, Eccles MP, Grimshaw JM (2010). What is an adequate sample size? Operationalising data saturation for theory-based interview studies. Psychol Health.

[23] D’Andrade RG, Quinn N (2005). Some methods for studying cultural cognitive structures. Finding culture in talk: a selection of methods.

[24] Hennink M, Hutter I, Bailey A (2011). Qualitative Research Methods.

[25] Miles MB, Huberman AM (1994). Qualitative Data Analysis: An Expanded Sourcebook.

[26] Bird J, Cohen-Cole SA (1990). The three-function model of the medical interview. An educational device. Adv Psychosom Med.

[27] Lipkin JM, Putnam SM, The LA, Interview M (1995). Clinical care, Education and Research.

[28] Grundmeijer HGLM, Reenders K, Rutten G (2009). Het geneeskundig proces. Klinisch redeneren van klacht naar therapie (in Dutch).

[29] Speckens AE, Van Hemert AM, Bolk JH, Rooijmans HG, Hengeveld MW (1996). Unexplained physical symptoms: outcome, utilization of medical care and associated factors. Psychol Med.

[30] Nimnuan C, Hotopf M, Wessely S (2001). Medically unexplained symptoms: an epidemiological study in seven specialities. J Psychosom Res.

[31] Salmon P (2007). Conflict, collusion or collaboration in consultations about medically unexplained symptoms: The need for a curriculum of medical explanation. Patient Educ Couns.

[32] Burton C (2003). Beyond somatisation: a review of the understanding and treatment of medically unexplained physical symptoms (MUPS). Br J Gen Pract.

[33] Deary IJ (1999). A taxonomy of medically unexplained symptoms. J Psychosom Res.

[34] Tickle-Degnen L, Rosenthal R (1990). The nature of rapport and its nonverbal correlates. Psychological Inquiry.

[35] Bernieri FJ. The Expression of Rapport. In: Manusov V, editor. Mahwah: Lawrence Erlbaum Associates Publishers; 2005 347–359

[36] Bernieri FJ, Gillis JS, Davis JM, Grahe JE (1996). Dyad rapport and the accuracy of its judgment across situations: A lens model analysis. J Pers Soc Psychol.

[37] Ramseyer F, Tschacher W (2011). Nonverbal Synchrony in Psychotherapy: Coordinated Body Movement Reflects Relationship Quality and Outcome. J Consult Clin Psychol.

[38] Martin DJ, Garske JP, Davis MK (2000). Relation of the therapeutic alliance with outcome and other variables: a meta-analytic review. J Consult Clin Psychol.

[39] Tickle-Degnen L, Gavett E, Philippot P, Feldman RS, Coats EJ (2003). Changes in nonverbal behavior during the development of therapeutic relationships. Nonverbal behavior in clinical settings.

[40] Epstein RM (2013). Whole mind and shared mind in clinical decision-making. Patient Educ Couns.

[41] Zwar N, Harris M, Griffiths R (2006). A Systematic Review of Chronic Disease management.

[42] Wagner EH, Bennett SM, Austin BT, Greene SM, Schaefer JK, Vonkorff M (2005). Finding common ground: patient-centeredness and evidence-based chronic illness care. J Altern Complement Med..

[43] Michie S, Miles J, Weinman J (2003). Patient-centredness in chronic illness: what is it and does it matter?. Patient Educ Couns.

[44] Dwamena F, Holmes-Rovner M, Gaulden CM, Jorgenson S, Sadigh G, Sikorskii A, Lewin S, Smith RC, Coffey J, Olomu A (2012). Interventions for providers to promote a patient-centred approach in clinical consultations. Cochrane Database Syst Rev..

[45] Hibbard JH, Greene J (2013). What the evidence shows about patient activation: better health outcomes and care experiences; fewer data on costs. Health Aff (Millwood).

[46] Beach MC, Inui T (2006). Relationship-centered care - A constructive reframing. J Gen Intern Med.

[47] Suchman AL (2006). A new theoretical foundation for relationship-centered care. Complex responsive processes of relating. J Gen Intern Med.

[48] Oprea L (2009). An analytic review of the doctor-patient relationship (Part II). Revista Romana de Bioetica.

[49] Silverman JD, Draper J, Kurtz SM (1995). The inhumanity of medicine. Interpersonal and communication skills can be taught. BMJ.

[50] Hilderink PH, Collard R, Rosmalen JG, Oude Voshaar RC (2015). How does ageing affect the impact of medically unexplained symptoms and medically explained symptoms on health-related quality of life?. Int J Geriatr Psychiatry.

[51] Isaac ML, Paauw DS (2014). Medically unexplained symptoms. Med Clin North Am.

[52] van der Feltz-Cornelis CM, Hoedeman R, Keuter EJ, Swinkels JA (2012). Presentation of the multidisciplinary guideline medically unexplained physical symptoms (MUPS) and somatoform disorder in the Netherlands: disease management according to risk profiles. J Psychosom Res.

[53] Hull M (2007). Building a rapport with patients. Foundation Years.

[54] van den Eertwegh V, van Dulmen S, van Dalen J, Scherpbier AJ, van der Vleuten CP (2013). Learning in context: Identifying gaps in research on the transfer of medical communication skills to the clinical workplace. Patient Educ Couns.

[55] Silverman J (2011). Clinical communication training in continuing medical education: possible, do-able and done?. Patient Educ Couns.

