# Supplementary material for: ‘It just has to click’: Internists’ views of: what constitutes productive interactions with chronically ill patients
Source: BMC Health Serv Res. 2016 May 27;16:191. doi: 10.1186/s12913-016-1430-6 (PMC4884358; doi:10.1186/s12913-016-1430-6)
Supplement: Additional file 1: — Interview topic list. (DOC 35 kb) [file 12913_2016_1430_MOESM1_ESM.doc]

**Additional file** to the manuscript titled: ‘It just has to click’: internists’ views of productive interactions with chronically ill patients. A qualitative study.

**Interview topic list for productive interaction**

1. What do you understand or define as a productive interaction?
2. What do you expect of (the outcome) a productive interaction?
3. What kind of (out)patients do you see in your consultation (room)?
4. Please describe the course of a conversation with your patients?

(*Do you talk about,* *e.g., what has happened; complaints and causes; medical history; medications; the patient’s ideas and or worries; psychological, social and family issues; lifestyle; organization of care; self-management; and compliance?*). What happens when this conversation does not go very well? Can you describe different types of situations and patients, and give examples?

1. What is important for achieving a productive interaction during your consultations?
2. (How) Do you involve/engage the patient? (*e.g., giving space to the patient’s: expectations, preferences and/or ideas about the cause of the illness; role in decision making and reaching agreement*)
3. What are conditions for a productive interaction? What (else) influences a productive interaction?*( E.g., environment, other persons, mutual bond/understanding, trust, patient characteristics; expertise, experience and other characteristics of the physician; complexity, seriousness and stage of the illness*)
4. What characterizes the relationship between patient and doctor in a productive interaction?
   (*E.g., a professional and/or caring and/or equal and/or personal relationship.*) What is the impact of this on the interaction? How do you view your role in this relationship? (*E.g., advisor, coach, parent, partner*). What are the influences of the complexity, seriousness and stage of the illness on your role?
5. How do you view the role of the internist regarding the coordination of care of patients with multiple problems?
6. What communication capacities/skills does a doctor need in order to reach a productive interaction? How do you assess your own skills? How do you practice these skills? What importance do you attach to communication skills?
