# Supplementary material for: ‘It just has to click’: Internists’ views of: what constitutes productive interactions with chronically ill patients
Source: BMC Health Serv Res. 2016 May 27;16:191. doi: 10.1186/s12913-016-1430-6 (PMC4884358; doi:10.1186/s12913-016-1430-6)
Supplement: Additional file 2: — Sample quotes for all goals. (DOCX 24 kb) [file 12913_2016_1430_MOESM2_ESM.docx]

**Additional file** to the manuscript titled: ‘It just has to click’: internists’ views of productive interactions with chronically ill patients. A qualitative study

Sample quotes from participants’ goal-directed descriptions of a productive interaction, divided into four types of goal orientations

| 1. Health outcome- oriented | 1. Satisfaction-oriented |
| --- | --- |
| 1. Treatment goals are achieved (risks reduced, complications prevented)   ‘A productive interaction is, in my terms, very much *targeted at the final product*, in particular good blood pressure, good average sugar regulation, and good cholesterol levels, and … this determines if you can measure whether something is productive or not.’ (subspec)   1. The wellbeing of the patient is protected  ‘*Quality of life is essential* and if your treatment reduces the quality of life to gain only a few weeks of life expectancy, *then someone will not really profit*; so I find that quality of life indeed important.’ (subspec) 2. The patient feels they are being helped with their problems   ‘Whether it [the consultation] is productive depends very much on *what the patient will be left with*…To me it is also about overlooking nothing and, to a patient, it is about *removing that ailment*, but with that I cannot always help them.’ (gen)   1. The patient functions better/independently  ‘When *the patient* acquires more information and is able to draw conclusions, and … *to take actions and be less dependent*, that is very productive.’ (subspec) | 1. Patient and physician both feel satisfied   ‘It is the collaboration between patient and doctor *that makes both leave the room feeling satisfied*. That is a real productive interaction. The basic condition for that is you are trying to help someone as much as it is medically possible and that the patient feels helped; that the patient has the idea that they have been heard and that you have the feeling that there has been an authentic contact and not merely an instrumental contact.’ (gen)   1. The patient feels satisfied   ‘Most important is that you determine what the patient wants, that you are dedicated to that, irrespective of whether it is entirely your discipline and whether you can solve it easily, and that *a patient leaves satisfied*, also in the long term.’ (gen)   1. The physician feels satisfied   ‘After a few months some patients say, now I can do it myself, *then I am really satisfied,* then I feel I get something in return. I have put something in, and now they are so well advanced that they are aware of what the problem is.’ (gen) |
| 1. Medical process-oriented | 1. Collaboration-oriented |
| 1. The (medical) puzzle is solved; medical causes are identified or ruled out   ‘Ideally, *you solve the patient’s problem* .., but that rarely happens. Still worse, in most cases, you *will not find a cause at all.’* (gen)   1. The patients’ problems or questions are understood   ‘A productive interaction is one where…a patient can find the right words for their motives or ailments, or where *I can see nonverbally something is the matter and that I can also clarify what the request for help really is.*’ (subspec)   1. Expert explanations and or advice are given   ‘Given my goal, it [a productive interaction] is where I have *offered advice to someone* and have said: ‘This seems to me the most sensible option’ and that I feel I have given good advice and, yes, then it is up to the patient … to hear that and to take that with them.’ (subspec)   1. The patient understands the (medical) explanations/advice   ‘A productive interaction is where *they understand* what I am going to do and when I have made a diagnosis, or not, what that means - and when I prescribe pills that they have to take them.’ (gen)   1. The physician works effectively or efficiently   ‘…actually you are practicing together and that is fun - but it does take much time, and thus it is not productive *in the sense of efficiency*; it takes considerable time, but on the other hand it is care that has to be provided.’ (subspec) | 1. The physician connects with the patient   ‘Often it [a productive interaction] *is the first contact*, and the attention and the listening that you put into it, that brings something to the following contacts …There you *build the important bond of trust*.’ (gen)   1. The physician and patient reach mutual understanding   ‘A productive interaction is one where you *create mutual openness* and you can understand each other in that way; not just hear, but really understand.’ (subspec)   1. The physician and the patient reach agreement   ‘A productive interaction is where you *reach consensus* about what the patient perceives as a problem, and then you explain what you will do to clarify that problem biomedically, and subsequently you communicate what you will do about it.’ (subspec) |
| Typical examples of each of the 15 goal-directed descriptions of a productive interaction identified in an analysis of 275 text fragments. The part of the fragment that particularly refers to the goal is shown in italics. Note that a quote can reflect more than one goal (e.g., the text in A3 also addresses the goal in C1 and vice versa).  (subspec) = quote from a subspecialist; (gen) = quote from a generalist. | |
